# Circulating MicroRNAs Related to Arterial Stiffness in Adults with HIV Infection

**DOI:** 10.3390/v16121945

**Published:** 2024-12-19

**Authors:** Sideris Nanoudis, Maria P. Yavropoulou, Olga Tsachouridou, Maria Pikilidou, Dimitrios Pilalas, Kalliopi Kotsa, Lemonia Skoura, Pantelis Zebekakis, Symeon Metallidis

**Affiliations:** 1 1st Internal Medicine Department, AHEPA University Hospital, School of Medicine, Aristotle University of Thessaloniki, 55436 Thessaloniki, Greece; sidnanoudis@yahoo.gr (S.N.); myavropoulou@med.uoa.gr (M.P.Y.); pikilidou@gmail.com (M.P.); dpilalas@auth.gr (D.P.); kkalli@auth.gr (K.K.); pzebeka@auth.gr (P.Z.); metallidissimeon@yahoo.gr (S.M.); 2 Department of Microbiology, AHEPA University Hospital, School of Medicine, Aristotle University of Thessaloniki, 55436 Thessaloniki, Greece; mollyskoura@gmail.com

**Keywords:** PWH, miRNA, arterial stiffness, cfPWV, cardiovascular risk

## Abstract

People with HIV (PWH) have an elevated risk of cardiovascular disease compared to those without HIV. This study aimed to investigate the relative serum expression of microRNAs (miRNAs) associated with arterial stiffness, a significant marker of cardiovascular disease. A total of 36 male PWH and 36 people without HIV, matched for age, body mass index, pack years, and dyslipidemia, were included in the study. Participants with a history of hypertension, diabetes mellitus, cardiovascular disease, cancer, or intravenous drug use were excluded. Markers of arterial stiffness, including carotid–femoral pulse wave velocity (cfPWV) and augmentation index adjusted to 75 beats per minute (AIx@75), were measured via applanation tonometry. We analyzed the relative expression of 11 circulating miRNAs using real-time PCR: let-7b-5p, miR-19b-3p, miR-21-5p, miR-29a-3p, miR-126-3p, miR-130a-3p, miR-145-5p, miR-181b-5p, miR-221-3p, miR-222-3p, and miR-223-3p. cfPWV was significantly higher in PWH compared to people without HIV (9.3 vs. 8.6 m/s, *p* = 0.019), while AIx@75, peripheral, and aortic blood pressures did not differ among groups. The relative expression of circulating miRNAs was significantly higher in PWH compared to controls for let-7b-5p (fold change: 5.24, *p* = 0.027), miR-21-5p (fold change: 3.41, *p* < 0.001), miR-126-3p (fold change: 1.23, *p* = 0.019), and miR-222-3p (fold change: 3.31, *p* = 0.002). Conversely, the relative expression of circulating miR-19b-3p was significantly lower in PWH (fold change: 0.61, *p* = 0.049). Among HIV-related factors, the nadir CD4+T-cell count of <200 cells/mm^3^ was independently associated with the relative expression of circulating let-7b-5p (β = 0.344, *p* = 0.049), while current non-nucleoside reverse transcriptase inhibitor (NNRTI) treatment was independently associated with the relative expression of circulating miR-126-3p (β = 0.389, *p* = 0.010). No associations were found between the duration of HIV infection or the duration of ART and the serum miRNA expression. This study highlights a distinct circulating miRNA profile in PWH with higher cfPWV compared to those without HIV, which may contribute to increased arterial stiffness.

## 1. Introduction

The introduction of highly active antiretroviral therapy (HAART) in 1996 significantly altered the progression and outcomes of human immunodeficiency virus (HIV) infection, leading to increased patient survival and a reduction in the development of AIDS (Acquired Immunodeficiency Syndrome) [1]. The majority of people with HIV (PWH) who have access to antiretroviral therapy (ART) are considered to have a manageable chronic condition, with a life expectancy and quality of life comparable to people without HIV [2]. According to the Centers for Disease Control and Prevention (CDC), more than half of PWH in the United States in 2022 were aged 50 and older [3]. However, aging with HIV is associated with a higher prevalence of non-AIDS comorbidities compared to people without HIV, including cardiovascular disease, renal failure, malignancies, and neurological disorders [4]. Persistent inflammation and chronic immune activation caused by the virus itself, as well as side effects of antiretroviral drugs, are strongly linked to non-AIDS related morbidity and mortality [4,5,6,7,8].

Arterial stiffness reflects normal vascular aging and is considered a significant predictor of cardiovascular disease. The stiffness of elastic arteries, particularly the aorta, is recognized as an independent risk factor for major cardiovascular events, including coronary artery disease, heart failure, and stroke [9,10]. Carotid–femoral pulse wave velocity (cfPWV) is regarded as the “gold standard” for non-invasive assessment of large artery stiffness [11]. According to the 2023 ESH Guidelines for the management of arterial hypertension, a cfPWV greater than 10 m/s indicates asymptomatic organ damage and serves as the standard threshold for predicting cardiovascular events [12]. Additionally, the aortic augmentation index (AIx) is considered a surrogate marker of arterial stiffness [13,14]. The impact of HIV infection on arterial stiffness remains a topic of debate. Numerous case–control studies have shown a positive association between HIV infection and increased cfPWV in age- and sex-matched groups, while others have found no significant differences [15,16]. However, most researchers agree that, in addition to traditional risk factors such as age, smoking, blood pressure, and body mass index, HIV-related factors—including chronic inflammation, CD4+T-cell counts <200 cells/mm^3^, nadir CD4+T-cell counts <200 cells/mm^3^, and metabolic disorders associated with ART, such as dyslipidemia and insulin resistance—may also contribute to accelerated arterial stiffening [17,18,19,20].

MicroRNAs (miRNAs) are small, non-coding RNAs, typically 19–25 nucleotides in length, that regulate gene expression post-transcriptionally. They suppress target gene expression by inhibiting translation and inducing degradation of their mRNA target sequences [21]. The first miRNA, lin-4, was discovered in 1993 [22]. Research into miRNAs has intensified over the past fifteen years due to their emerging roles in various diseases and biological processes, including atherosclerosis, cardiovascular disease, cancer, and diabetes mellitus [23,24]. MiRNAs influence arterial stiffness by targeting key pathways and mechanisms related to extracellular matrix (ECM) composition and endothelial dysfunction [25,26]. While many studies focus on miRNAs within the cell microenvironment, it is also well established that they are present in biological fluids such as serum, plasma, saliva, and cerebrospinal fluid, where they can effectively reflect tissue changes. MiRNAs from various cell types can be secreted into the extracellular space and transported to circulating body fluids, such as peripheral blood, where they exhibit remarkable stability. This stability is attributed to their encapsulation in extracellular microvesicles, binding with lipid proteins, or association with protective complexes, making them resistant to RNase digestion and other degrading factors such as extreme temperatures and pH changes [27,28,29].

The aim of this study is to compare the relative expression of circulating miRNAs associated with arterial stiffness between PWH and people without HIV. For this purpose, we measured the relative serum expression of let-7b-5p, miR-19b-3p, miR-21-5p, miR-29a-3p, miR-126-3p, miR-130a-3p, miR-145-5p, miR-181b-5p, miR-221-3p, miR-222-3p, and miR-223-3p in both groups. The selection of miRNAs was based on existing literature identifying specific miRNAs correlated with mechanisms of arterial stiffness in both serum and tissue samples (Table 1). Additionally, we searched the following databases to identify biological targets of miRNAs in humans: (a) TargetScan, (b) miRDB, and (c) miRTarBase. We focused on 8mer, 7mer, and 6mer sites that match the seed region for each miRNA, prioritizing conserved sites and the best cumulative scores.

## 2. Materials and Methods

### 2.1. Study Population

This study is a cross-sectional, case–control study focused on exploring the potential impact of HIV infection and ART on arterial stiffness and miRNA expression. We compared 36 male PWH for whom it had been at least five years since their initial confirmatory HIV test, with 36 male people without HIV (control group). All participants were over 48 years old and had no history of hypertension, diabetes mellitus, or cardiovascular disease. We excluded individuals with neoplastic disease, cirrhosis, chronic kidney disease stage 4 or 5 (estimated glomerular filtration rate [eGFR] <30 mL/min/1.73 m^2^, as calculated using the CKD-EPI equation), and intravenous drug use. The two groups were matched for age, body mass index (BMI), pack years, and dyslipidemia in order to investigate whether HIV infection independently affects arterial stiffness. PWH were recruited from the outpatient department of our hospital (AIDS Excellence Centre of Northern Greece, AHEPA General University Hospital of Thessaloniki), while the control subjects volunteered in response to a local announcement. Written informed consent was obtained from all subjects involved in the study. The study was conducted in accordance with the Declaration of Helsinki, and the protocol was approved by the local Ethics Committee under protocol number 1.88/2018.

### 2.2. Demographic and Clinical Data

Sociodemographic characteristics (age, gender, race, profession), medical history, and concomitant medication of each participant were recorded using a questionnaire. Participants were classified as hypertensive and excluded from the study if they had a known history of hypertension and/or were receiving antihypertensive treatment. Similarly, individuals were considered as diabetic and excluded from the study if they had a known history of diabetes mellitus, were receiving treatment for diabetes, and/or had a fasting plasma glucose level >126 mg/dl. Cardiovascular disease was defined as the presence of any of the following conditions: coronary artery disease, chronic heart failure, peripheral artery disease, or cerebrovascular disease. A history of smoking, pack years, alcohol abuse, and physical activity were also documented. HIV medical history, including duration of HIV infection, duration and type of ART regimens, and nadir CD4+T-cell count, was obtained from PWH. A history of AIDS-defining conditions within the past three years was also an exclusion criterion.

### 2.3. Physical Measurements

A physical examination was conducted for all participants, including measurements of weight, height, waist circumference, heart rate, and brachial blood pressure. BMI was calculated by dividing body mass (kg) by the square of height (m^2^). Participants were seated for five minutes in a quiet room before blood pressure measurements were taken. Blood pressure was measured in the sitting position three times, with two-minute intervals between measurements, on both upper arms (brachial artery) using an oscillometric automatic sphygmomanometer (Microlife BP A6 PC, Microlife Corporation, Taipei, Taiwan) with an appropriately sized arm bladder and cuff, in accordance with the 2023 ESH Guidelines for the management of arterial hypertension [12]. The average of the second and third measurements from both arms was calculated, and the higher average value was recorded as the reference brachial blood pressure.

### 2.4. Laboratory Samples

Blood samples were collected from all subjects after a 9 h fasting period for routine testing and miRNA analysis at local labs (Microbiology Department and Division of Endocrinology and Metabolism, AHEPA General University Hospital of Thessaloniki). Tests included a complete blood cell count and measurements of glucose, urea, creatinine, and a lipid profile (total cholesterol, high-density lipoprotein cholesterol [HDL-C], low-density lipoprotein cholesterol [LDL-C], and triglycerides). A history of dyslipidemia was defined as LDL-C > 115 mg/dl and/or lipid-lowering drug treatment. CD4+T-cell count and viral load were also measured in PWH. eGFR was calculated using the Chronic Kidney Disease Epidemiology Collaboration (CKD-EPI) equation. To assess total cardiovascular risk, we used the Framingham Risk Score (FRS) and the Systemic Coronary Risk Estimation (SCORE) system adjusted for Greece (HellenicSCORE II), according to 2019 ESC/EAS Guidelines for the Management of Dyslipidemias and the 2023 updated guidelines of the Hellenic Atherosclerosis Society [59,60]. HellenicSCORE II estimates the 10-year risk of fatal cardiovascular events, including sudden cardiac death, based on gender, age, smoking status, peripheral systolic blood pressure, and total cholesterol. Additionally, we used the risk score from the data collection on adverse events of anti-HIV drugs (D:A:D) to estimate the 5-year risk of cardiovascular events in people with HIV.

### 2.5. Pulse Wave Velocity and Pulse Wave Analysis

Measurements of cfPWV and central aortic blood pressures were obtained using applanation tonometry with a SphygmoCor device (AtCor, Sydney, Australia). cfPWV was calculated by dividing the distance between the two recording sites by the transit time. The cfPWV measurement was performed by recording pressure waveforms using a hand-held tonometer (SPT-301, Millar Inc., Houston, TX, USA) at the common carotid artery and then at the common femoral artery, with a simultaneously recorded electrocardiogram signal. The waveforms were captured by placing the tonometer on the skin over the strongest pulse points along the common carotid and common femoral arteries, ensuring a minimum of 10 s of high-quality waveforms. The distance between the common carotid and common femoral pulse points was measured in a straight line with a tape measure, and 80% of this distance was used to determine the pulse wave travel distance, in accordance with the 2012 “Expert Consensus Document on the Measurement of Aortic Stiffness in Daily Practice Using Carotid-Femoral Pulse Wave Velocity” [11]. Pulse wave travel time was automatically calculated by the device based on the time delay of the R-wave of the electrocardiogram signal between the common carotid and common femoral pressure waveforms. All measurements were performed by the same trained physician in a quiet room with the subject in a supine position after 15 min of rest. Subjects were instructed not to smoke, eat, or drink coffee within 4 h before their visit. The average of two valid measurements was used as the reference for cfPWV.

Central aortic systolic blood pressure (aSBP) and central aortic diastolic blood pressure (aDBP) were derived from pressure waveforms recorded at the radial artery, which were calibrated using brachial systolic and diastolic blood pressure. Measurements were taken by placing the tonometer over the strongest pulse point of the radial artery and recording a minimum of 10 s of consistent radial waveforms. The average of two valid measurements was used as the reference for aSBP and aDBP. The augmentation index, corrected to a heart rate of 75 bpm (AIx@75), was calculated using the formula: (augmentation pressure)/(central pulse pressure). Augmentation pressure is the difference between aSBP and the inflection pressure, while central pulse pressure is the difference between aSBP and aDBP.

### 2.6. MiRNA Analysis

#### 2.6.1. Sample Processing

Whole blood was collected in tubes containing a clot activator and left at room temperature for at least 10 min, but no longer than 1 h, before centrifugation. The samples were centrifuged for 10 min at 1900× *g* and 4 °C. The serum phase was then transferred to conical tubes and subjected to a second centrifugation for 10 min at 16,000× *g* and 4 °C in a fixed-angle rotor to remove additional cellular nucleic acids attached to cell debris. The serum was aliquoted and frozen at –80 °C until further processing. For processing frozen lysates, samples were incubated in a 37 °C water bath until completely thawed, and salts were dissolved. After thawing, serum samples were centrifuged for 5 min at 16,000× *g* and 4 °C to remove cryoprecipitates.

#### 2.6.2. Isolation of MiRNA from the Serum

RNA was extracted from 200 µL of serum sample using the miRNeasy Serum/Plasma Kit, following the manufacturer’s instructions (Qiagen, Hilden, Germany). During the purification process, a synthetic RNA sequence (spike-in control: Caenorhabditis elegans cel-miR-39-3p) was added in an appropriate amount to serum preparations after homogenization with QIAzol lysis reagent to control for variations in recovery and amplification efficiency between RNA preparations. Additionally, 1.25 mg/mL bacteriophage MS2 RNA was used as a carrier. Total RNA was eluted with 20 µL of RNase-free water.

#### 2.6.3. Reverse Transcription and PCR Analysis

After elution, 1.5 µL of the RNA sample was used as a template for reverse transcription with the miScript II RT Kit (Qiagen) using the miScript HiSpec Buffer. Upon completion of the reverse transcription reaction, each sample was diluted to 220 µL with RNase-free water. Subsequently, 2 µL of the diluted reaction was used as a template for each single miRNA assay, following the manufacturer’s instructions (Qiagen, Hilden, Germany). Cycling was performed under standardized conditions using the miScript SYBR Green PCR Kits on the QIAGEN Rotor-Gene Q (Corbett Rotor-Gene 6000) real-time PCR cycler.

#### 2.6.4. Selection of MiRNA Primer Assays

The following miScript Primer Assays were used for the selected miRNAs: (1) MS00003122 for hsa-let-7b-5p, (2) MS00006545 for hsa-miR-19b-3p, (3) MS00003213 for hsa-miR-21-5p, (4) MS00044653 for hsa-miR-29a-3p, (5) MS00003430 for hsa-miR-126-3p, (6) MS00003444 for hsa-miR-130a-3p, (7) MS00003528 for hsa-miR-145-5p, (8) MS00006699 for hsa-miR-181b-5p, (9) MS00003857 for hsa-miR-221-3p, (10) MS00007609 for hsa-miR-222-3p, and (11) MS00003871 for hsa-miR-223-3p (Table 1). hsa-miR-16-5p (MS00006517) and miR-451a (MS00004242) were used as normalization controls for variability in sample loading and real-time RT-PCR efficiency. All reactions were performed in duplicate.

Mean Ct values from quantitative RT-PCR analysis were uploaded to the Qiagen website for analysis (GeneGlobe Data Analysis Center, miScript Primer Assays). The Ct cut-off for the lower limit of detection was set at 33, as recommended by the software instructions. Lower Ct values indicate higher levels of miRNA expression. ΔCt values were calculated, and relative normalized gene expression in each group was determined using the 2^−ΔCt^ method. Fold change (2^−ΔΔCt^) in relative miRNA expression between groups was used for further analysis, defined as the normalized gene expression in PWH divided by the normalized gene expression in the control group. Fold-change values less than one indicated down-regulation, while values greater than one indicated up-regulation of relative gene expression. *p*-values were calculated using a Student’s I-test on the replicate 2^−ΔCt^ values for each gene in the study groups. *p*-values less than 0.05 were considered statistically significant.

#### 2.6.5. Quality Control

To assess the efficiency of RNA extractions and to detect any potential inhibitors in cDNA synthesis or PCR, we quantified the levels of the spike-in control added before RNA extraction using real-time PCR. All cDNAs expressed the spike-in control at normal levels, with Ct values less than 24 cycles. Statistical analysis confirmed the absence of outliers in the Ct values for the spike-in control. All genes of interest were successfully expressed in the study population.

### 2.7. Statistical Analysis

The Shapiro–Wilk test was used to assess the normality of distribution. Data are reported as mean ± SD or median (25th–75th percentile) for continuous variables and as number and percentages for categorical variables. Student’s *t*-test and the Mann–Whitney U-test were employed to compare the means of normally distributed and asymmetrically distributed variables, respectively. The chi-square (χ2) test was used to examine differences between categorical data. Pearson correlation or Spearman’s rank-order correlation was performed to determine the relationships between markers of arterial stiffness and cardiovascular risk factors, as well as between differentially expressed miRNAs and HIV-related characteristics, as applicable. Simple linear and multivariable regression analyses were used to investigate associations between differentially expressed miRNAs and variables of interest. Variables with a *p*-value < 0.05 were included in the multiple linear regression analysis. Receiver operating characteristic (ROC) analysis was used to assess the sensitivity and specificity of relative miRNA expression in PWH with increased arterial stiffness. All tests were two-sided, and a *p*-value < 0.05 was considered statistically significant. Statistical analysis was conducted using IBM SPSS Statistics for Windows, version 26.0 (IBM Corporation, Armonk, NY, USA).

## 3. Results

The clinical and laboratory characteristics as well as arterial wall parameters for both groups are presented in Table 2. There were no statistically significant differences between PWH and people without HIV in terms of age, BMI, smoking status, dyslipidemia, renal function, peripheral blood pressure, HellenicSCORE II, or Framingham Risk Score (FRS). HDL-C was significantly higher in people without HIV (53 vs. 45 mg/dl, *p* = 0.002), but other lipid and glucose metrics did not differ significantly between the study groups. cfPWV was higher in PWH compared to people without HIV (9.3 vs. 8.6 m/s, *p* = 0.019). AIx@75, CSBP, and CDBP were also higher in PWH, though these differences did not reach statistical significance.

HIV-specific characteristics are detailed in Table 3. The median duration of HIV infection was 13 years (range: 8 to 19.8 years). All participants were on ART, with a median duration of 12 years (range: 7 to 18 years), and 97.2% were virologically suppressed. The class and duration of each ART regimen are also presented in Table 3.

Differences in circulating miRNA expression profiling between groups are presented in Table 4. The relative expression of circulating miRNAs (2^−ΔCt^) was significantly increased in PWH compared to controls for let-7b-5p (*p* = 0.027), miR-21-5p (*p* < 0.001), miR-126-3p (*p* = 0.019), and miR-222-3p (*p* = 0.002). Conversely, the relative expression of circulating miR-19b-3p was significantly lower in PWH (*p* = 0.049). No significant differences were observed between groups for miR-29a-3p, miR-130a-3p, miR-145-5p, miR-181b-5p, miR-221-3p, or miR-223-3p. The fold change (2^−ΔΔCt^) in PWH compared to people without HIV was 5.24 (1.86~8.61) for let-7b-5p, 0.61 (0.20~1.01) for miR-19b-3p, 3.41 (1.75~5.07) for miR-21-5p, 1.23 (0.64~1.82) for miR-126-3p, and 3.31 (1.10~5.51) for miR-222-3p.

**Figure 1:**
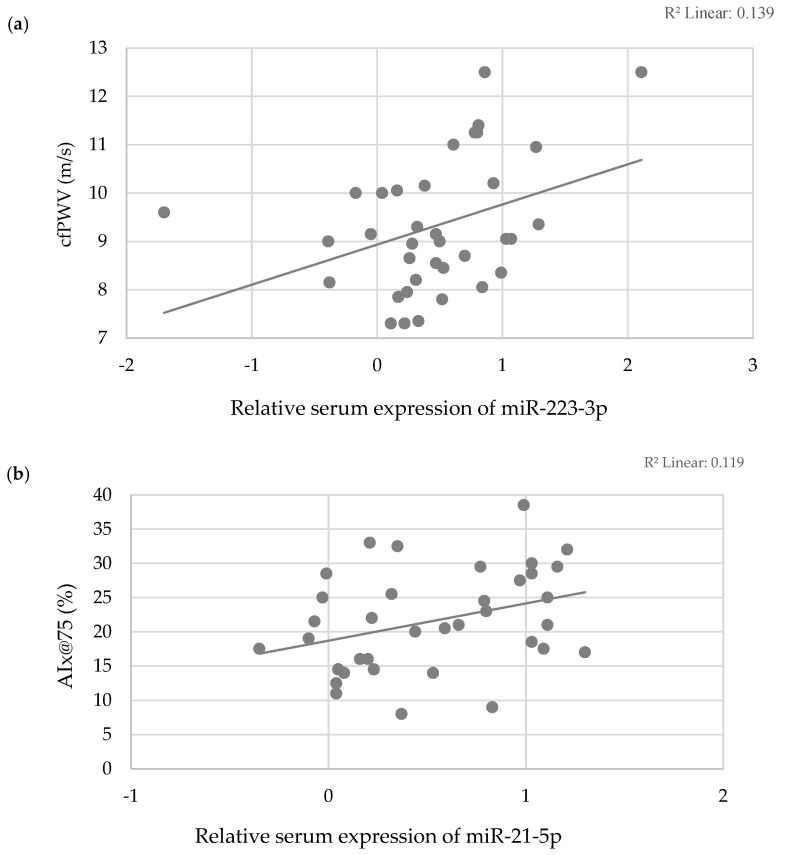
Scatter plots illustrating correlations between (**a**) cfPWV and relative serum expression of miR-223-3p and (**b**) AIx@75 and relative serum expression of miR-21-5p.

Further analysis within PWH revealed a significant correlation between cfPWV and age (ρ = 0.429, *p* = 0.009), brachial systolic blood pressure (r = 0.374, *p* = 0.025), HellenicSCORE II (ρ = 0.436, *p* = 0.008), FRS (r = 0.469, *p* = 0.004), D:A:D SCORE (r = 0.364, *p* = 0.029), and the relative serum expression of miR-223-3p (r = 0.373, *p* = 0.025). Similarly, AΙx@75 was associated with age (ρ = 0.465, *p* = 0.004), brachial diastolic blood pressure (r = 0.453, *p* = 0.006), HellenicSCORE II (ρ = 0.337, *p* = 0.044), and the relative serum expression of miR-21-5p (r = 0.344, *p* = 0.040) (Figure 1).

Linear regression models were utilized to investigate the relationships between differentially expressed miRNAs and clinical, laboratory, hemodynamic, and HIV-related characteristics. No correlations were found between the duration of HIV infection or the duration of ART and miRNA relative expression levels. In simple linear regression analysis, the relative expression of let-7b-5p was associated with brachial systolic blood pressure (β = 0.027, *p* = 0.001), brachial pulse pressure (β = 0.037, *p* = 0.002), nadir CD4+T-cell <200 cells/mm^3^ (β = 0.412, *p* = 0.046), and current tenofovir disoproxil (TDF) treatment (β = −0.519, *p* = 0.016) (Table 5).

The relative expression of miR-19b-3p showed a significant negative relationship with a history of smoking (β = −0.353, *p* = 0.032). Aside from AIx@75, the relative expression of miR-21-5p was associated with age (β = 0.033, *p* = 0.029) and brachial and aortic blood pressures.

Additionally, the relative expression of miR-126-3p was positively associated with age (β = 0.037, *p* = 0.029), brachial and aortic blood pressures, and current NNRTI treatment (β = 0.402, *p* = 0.025) (Table 6), while showing a negative correlation with eGFR (β = −0.014, *p* = 0.007). Finally, the relative expression of miR-222-3p did not show any correlation with these factors.

In multivariable models, nadir CD4+T-cell <200 cells/mm^3^ (β = 0.344, *p* = 0.049) and brachial pulse pressure (β = 0.030, *p* = 0.009) were identified as independent predictors of the relative expression of let-7b-5p, with an adjusted R^2^ of 0.340 (Table 5). Additionally, age (β = 0.032, *p* = 0.027), eGFR (β = −0.012, *p* = 0.004), and current NNRTI treatment (β = 0.389, *p* = 0.010) were independent predictors of the relative expression of miR-126-3p, with an adjusted R^2^ of 0.467 (Table 6).

**Figure 2:**
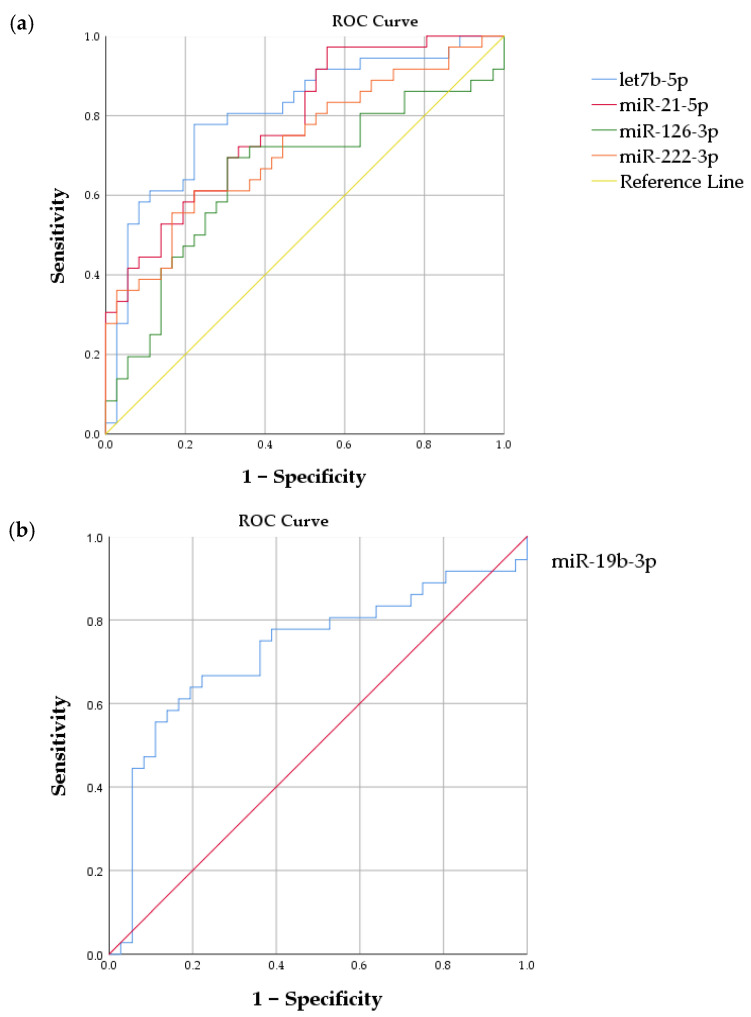
Receiver operating characteristic (ROC) curves for differentially expressed miRNAs let-7b-5p, miR-21-5p, miR-126-3p, miR-222-3p (**a**) and miR-19b-3p (**b**) in PWH with higher cfPWV.

AUCs were calculated for each differentially expressed circulating microRNA to evaluate the diagnostic accuracy of their relative expression in distinguishing between PWH with increased arterial stiffness and people without HIV with lower arterial stiffness (Figure 2). The AUC for let-7b-5p was 0.806 (95% CI: 0.703–0.909), with a sensitivity of 77.8% and specificity of 75%. The AUC for miR-21-5p was 0.780 (95% CI: 0.676–0.884), with a sensitivity of 72.2% and specificity of 66.7%. The AUC for miR-19b-3p was 0.725 (95% CI: 0.602–0.849), with a sensitivity of 75% and specificity of 63.9%. For miR-222-3p, the AUC was 0.725 (95% CI: 0.608–0.841), with a sensitivity and specificity of 63.9%. Lastly, the AUC for miR-126-3p was 0.654 (95% CI: 0.523–0.784), with both sensitivity and specificity at 69.4%.

## 4. Discussion

In this study, we demonstrated a differential expression of circulating microRNAs associated with mechanisms of arterial stiffness in PWH with higher cfPWV compared to people without HIV and lower cfPWV. We found significantly higher relative expression of let-7b-5p, miR-21-5p, miR-126-3p, and miR-222-3p, along with lower relative expression of miR-19b-3p. Although the duration of HIV infection and the duration of ART did not correlate with the relative expression of the microRNA panel, nadir CD4+T-cell count <200 cells/mm^3^ and current NNRTI treatment were independently associated with relative expression of let-7b-5p and miR-126-3p, respectively.

MicroRNAs are small, non-coding RNAs that regulate gene expression post-transcriptionally. Their crucial role in the development of various diseases has spurred extensive research into their potential as diagnostic markers and novel therapeutic targets. Research in miRNAs has particularly advanced in cancer, cardiovascular disease, and also HIV infection [21,23,61]. Their involvement in arterial stiffness has also been investigated, revealing that aberrant expression of microRNAs can influence specific molecular pathways [25,26]. Additionally, circulating miRNAs are considered promising biomarkers due to their high stability, ability to reflect tissue-level changes, and ease of extraction from blood samples [27,28,29]. Their high specificity and sensitivity have established them as biomarkers for diffuse large B-cell lymphoma since 2008 [62,63].

Increased stiffness of large arteries reflects vascular aging and is independently associated with morbidity and mortality from cardiovascular diseases [9,10]. It is well established that PWH are at a higher risk for cardiovascular disease; however, the relationship between HIV infection and arterial stiffness remains controversial [6,15,16,64]. Chronic inflammation, persistent immune activation, and the effect of ART are linked to endothelial dysfunction, vessel wall damage, extracellular matrix remodeling, angiotensin-II-mediated fibrosis, vascular smooth muscle cell (VSMC) dysfunction, and calcifications, all of which can contribute to elevated arterial stiffness and premature vascular aging [17,18,19,20,65,66].

In our study, we observed that PWH had significantly higher cfPWV compared to people without HIV. cfPWV is widely regarded as the gold-standard method for evaluating arterial stiffness [11]. Both groups were matched for age, BMI, pack years, and dyslipidemia. Additionally, participants with a history of any cardiovascular event, hypertension, or diabetes were excluded to eliminate confounding factors that could cause functional and structural damage to the arterial wall, leading to decreased vascular compliance. We then investigated circulating microRNAs that target genes related to the major pathways of arterial stiffness. Our research hypothesis posits that the altered relative expression of these miRNAs may be associated with increased arterial stiffness in PWH. The relative serum expression of let-7b-5p, miR-21-5p, miR-126-3p, and miR-222-3p was significantly higher in PWH, while the expression of miR-19b-3p was significantly lower.

Let-7b is highly expressed in various cardiovascular diseases, including cardiac fibrosis, atherosclerosis, heart failure, and ischemic stroke [67,68,69]. Additionally, let-7b-5p reduces both the number and functionality of endothelial colony-forming cells, promoting vascular cell senescence and apoptosis, as well as phenotypic changes in vascular smooth muscle cells by downregulating Hmga2 (High Mobility Group At-Hook 2) [32]. These mechanisms contribute to arterial stiffness and accelerated vascular aging. Our study findings suggest that past profound immunodeficiency (nadir CD4+T-cell <200 cells/mm^3^) in PWH may cause a predisposition to increased arterial stiffness due to its association with elevated levels of let-7b-5p. In contrast, treatment with TDF seems to be associated with lower levels of let-7b-5p; however, its effect on the variability of relative expression of let-7b-5p is less pronounced than that of severe immunosuppression.

miR-19b regulates extracellular matrix composition by directly targeting connective tissue growth factor (CTGF) and thrombospondin-1 (TSP-1). Low levels of miR-19b promote ECM remodeling by increasing the expression of CTGF and TSP-1, leading to tissue fibrosis [33,34,35]. Additionally, inhibition of miR-19b enhances endothelial cell apoptosis by modulating the Apaf1/caspase-dependent pathway [70]. In a study by Beaumont et al., decreased myocardial and serum levels of miR-19b in patients with aortic stenosis and heart failure were associated with increased left ventricular stiffness, possibly due to overexpression of the enzyme lysyl oxidase (LOX), which affects the collagen network [36]. In our study, we found that the relative expression of miR-19b-3p in serum was reduced in PWH. One or more of the aforementioned pathophysiological mechanisms may contribute to the increased arterial stiffness observed in this group compared to individuals without HIV. However, the relative expression of miR-19b-3p in serum among PWH did not correlate with any characteristics of HIV infection.

miR-21 is highly expressed in the cardiovascular system. In a previous Greek study, Parthenakis et al. reported that low levels of circulating miR-21 were associated with reduced arterial stiffness in patients with well-controlled hypertension [71]. In our study, the relative expression of circulating miR-21-5p was found to be elevated in PWH who exhibited increased arterial stiffness compared to people without HIV and reduced arterial stiffness. Furthermore, Lorenzen et al. demonstrated that miR-21 promotes fibrosis through an Ang II-mediated pathway by downregulating the anti-fibrotic targets PTEN and SMAD7 [37]. Similarly, Zhu et al. showed that miR-21 positively regulates the expression of collagen types I, III, and IV in human umbilical vein endothelial cells (HUVECs) via the TGF-β/SMAD3 pathway [72]. Another study found that miR-21 decreases the proliferation of endothelial progenitor cells by activating the TGF-β signaling pathway through downregulation of WW domain-containing protein 1 (WWP1) [38]. Finally, miR-21 promotes VSMC proliferation and migration by regulating both the hypoxia-inducible factor-1α/miR-21/tropomyosin 1 and Notch2/Jag-1 pathways [73]. The phenotypic switch of VSMCs from a contractile to a synthetic phenotype induces VSMC proliferation and extracellular matrix (ECM) accumulation, playing a major role in arterial stiffness [10,74,75].

On the other hand, overexpression of miR-126 seems to be beneficial for the vasculature, demonstrating anti-atherosclerotic and anti-inflammatory effects [76,77]. miR-126 reduces arterial stiffness and modulates vascular inflammation by suppressing vascular cell adhesion molecule 1 (VCAM-1) and attenuating collagen expression through the inactivation of the PI3K/AKT signaling pathway [43,44]. However, elevated levels of miR-126 have been observed in patients with vasculopathy, including hypertension, albuminuria, and Turner syndrome, which may be attributed to a compensatory mechanism in response to vascular damage [78,79,80,81,82]. In this context, the increased relative expression of circulating miR-126-3p could be interpreted similarly in our study. Additionally, current NNRTI treatment appears to exert a favorable effect in this regard, as it was independently associated with miR-126-3p levels.

Finally, miR-222-3p contributes to arterial stiffness by inducing phenotypic changes in vascular smooth muscle cells and promoting vascular calcification [25,83]. Liu and colleagues showed that inhibiting miR-221 and miR-222 in VSMC cultures reduces VSMC proliferation. This finding was confirmed in vivo, where inhibition of these miRNAs decreased VSMC proliferation and migration in rat carotid arteries. The identified target genes were p27 (Kip1) and p57 (Kip2) [52]. Similarly, another study showed that miR-222 promotes pulmonary arterial smooth muscle cell proliferation by targeting p27 and TIMP3 [55]. Additionally, Mackenzie et al. reported that miR-221 and miR-222 enhance arterial calcification through altered expression of Enpp1 and Pit-1 [53]. Typically, miR-221 and miR-222 act synergistically at the cellular level [84]. However, in our study, only miR-222-3p demonstrated a statistically significant difference in relative serum expression between the two groups. Consistent with miR-19b-3p and miR-21-5p, miR-222-3p showed no association with any HIV-related characteristics.

The most significant finding in our study was the observation of significantly higher relative expression of circulating let-7b-5p, miR-21-5p, miR-126-3p, and miR-222-3p, along with significantly lower relative expression of circulating miR-19b-3p in PWH compared to people without HIV. This altered miRNA expression may contribute to arterial stiffness through various molecular pathways, including ECM remodeling, VSMC proliferation, endothelial dysfunction, and arterial calcification. We hypothesize that the significantly higher cfPWV, a marker of arterial stiffness, observed in people with HIV may be partially attributed to the aberrant expression of these microRNAs. Additionally, we propose that there may be an epigenetic effect related to HIV infection that influences arterial stiffness through altered microRNA expression profiles. This hypothesis is further supported by the fact that both groups were matched for age, BMI, pack years, and dyslipidemia, with no history of hypertension, diabetes, or other cardiovascular diseases.

In addition, nadir CD4+T-cell <200 cells/mm^3^ were identified as an independent predictor of relative expression of circulating let-7b-5p, suggesting that past profound immunosuppression may accelerate arterial stiffness through the upregulation of let-7b-5p. The higher relative expression of circulating miR-126-3p in PWH could serve as a countermeasure against vascular damage associated with increased arterial stiffness, as was previously suggested in the literature [79,85]. Finally, ROC analysis indicated that circulating let-7b-5p, miR-19b-3p, miR-21-5p, miR-126-3p, and miR-222-3p demonstrate high diagnostic accuracy in differentiating between PWH with increased arterial stiffness and people without HIV with lower arterial stiffness.

Our study has several limitations that should be acknowledged. Firstly, we enrolled only male participants, as they represent the majority of PWH in our department. Additionally, the sample size was relatively small, underscoring the need for studies with larger populations, including women, to confirm our findings. This study is an observational cross-sectional study, which carries inherent limitations, such as the inability to capture variability in outcomes over time and the difficulty in establishing causality. Furthermore, since miRNAs have multiple target genes, other unaccounted factors may influence their expression. Additional prospective clinical studies are needed to investigate changes in relative miRNA expression over time and clarify the causal associations between their differential expression and markers of arterial stiffness, subclinical atherosclerosis, and HIV-related factors. Lastly, while we focused on a specific panel of microRNAs linked to mechanisms of arterial stiffness, employing a PCR array method could enable the identification of altered expression in a broader range of microRNAs.

## 5. Conclusions

In conclusion, this study presents preliminary data on the potential role of circulating microRNAs in increasing arterial stiffness in PWH. We found that the relative expression of circulating let-7b-5p, miR-21-5p, miR-126-3p, and miR-222-3p was upregulated, while the relative expression of circulating miR-19b-3p was downregulated in PWH, who exhibited significantly higher markers of arterial stiffness compared to people without HIV and lower cfPWV. Additionally, past profound immunodeficiency was independently associated with the relative expression of let-7b-5p. To the best of our knowledge, this is the first study to evaluate the role of microRNAs in arterial stiffness within PWH. Our findings suggest the potential application of these microRNAs as novel diagnostic or prognostic biomarkers and therapeutic targets, underscoring the need for further research in this area.

## Figures and Tables

**Table 1:** Selected miRNAs related to mechanisms of arterial stiffness.

Gene Symbol	MiScript Primer Assay Catalog	MiRNA Sequence	Target Gene	Predicted Function
hsa-let-7b-5p	MS00003122	5′UGAGGUAGUAGGUUGUGUGGUU	Hmga2	Increases arterial stiffness and promotes endothelial dysfunction [30].
hsa-miR-19b-3p	MS00006545	5′UGUGCAAAUCCAUGCAAAACUGA	CTGF, TSP-1, LOX	Decreases arterial stiffness [31,32,33,34].
hsa-miR-21-5p	MS00003213	5′UAGCUUAUCAGACUGAUGUUGA	PTEN/SMAD7, WWP1	Increases arterial stiffness [35,36].
hsa-miR-29a-3p	MS00044653	5′UAGCACCAUCUGAAAUCGGUUA	COL1A1, COL3A1, COL4A1, TGFB2, MMP2	Decreases arterial stiffness and arterial calcification [37,38,39,40].
hsa-miR-126-3p	MS00003430	5′UCGUACCGUGAGUAAUAAUGCG	VCAM-1, PI3K/AKT	Decreases arterial stiffness [41,42].
hsa-miR-130a-3p	MS00003444	5′CAGUGCAAUGUUAAAAGGGCAU	TGFBR1, ATG2B, RUNX3	Inhibits VSMC proliferation, maintains normal endothelial function, and reduces fibrosis [43,44,45].
hsa-miR-145-5p	MS00003528	5′GUCCAGUUUUCCCAGGAAUCCCU	TGFBR2, KLF4, CD40	Decreases arterial stiffness and VSMC proliferation [46,47,48].
hsa-miR-181b-5p	MS00006699	5′AACAUUCAUUGCUGUCGGUGGGU	TGFBi	Decreases arterial stiffness [49].
hsa-miR-221-3p	MS00003857	5′AGCUACAUUGUCUGCUGGGUUUC	p27, p57, Enpp1 and Pit-1, TIMP3	Promotes VSMC proliferation and arterial calcification [50,51,52]
hsa-miR-222-3p	MS00007609	5′AGCUACAUCUGGCUACUGGGU	p27, p57, Enpp1 and Pit-1, TIMP3	Promotes VSMC proliferation and arterial calcification [50,51,53]
hsa-miR-223-3p	MS00003871	5′UGUCAGUUUGUCAAAUACCCCA	ICAM-1, IL6/STAT3, IGF1R, mef2c	Decreases arterial inflammation and calcification, controversy on VSMC function [54,55,56,57,58].
cel-miR-39-3p	MS00019789	5′UCACCGGGUGUAAAUCAGCUUG	(-)	Spike-in control
hsa-miR-16-5p	MS00006517	5′UAGCAGCACGUAAAUAUUGGCG	(-)	Control housekeeping gene
hsa-miR-451a	MS00004242	5′AAACCGUUACCAUUACUGAGUU	(-)	Control housekeeping gene

hsa, homo sapiens; let, lethal, miR, microRNA; Hmga2, high mobility group protein 2; CTGF, connective tissue growth factor; TSP-1, thrombospondin-1; lysyl oxidase; PTEN, phosphatase and tensin homolog; SMAD7, mothers against decapentaplegic homolog 7; WWP1, WW domain-containing E3 ubiquitin protein ligase 1; COL1A1, collagen type I alpha 1; COL3A1, collagen type III alpha 1; COL4A1, collagen type IV alpha 1; TGFB2, transforming growth factor beta 2; MMP2, matrix metallopeptidase 2; VCAM-1, vascular cellular adhesion molecule-1; PI3K/AKT, phosphoinositide 3-kinase/protein kinase B; TGFBR1, transforming growth factor-beta receptor 1; ATG2B, autophagy-related 2B; RUNX3, runt-related transcription factor-3; TGFBR2, transforming growth factor-beta receptor 2; KLF4, Kruppel-like factor 4; CD40, Cluster of differentiation 40; TGFBi, transforming growth factor-beta-induced; Enpp1, ectonucleotide pyrophosphatase/phosphodiesterase family member 1; TIMP3, tissue inhibitor of metalloproteinase 3; ICAM-1, intercellular adhesion molecule-1; IL6, interleukin 6; STAT3, Signal transducer and activator of transcription 3; IGF1R, insulin-like growth factor 1 receptor; mef2c, myocyte-specific enhancer factor 2C; cel, Caenorhabditis elegans.

**Table 2:** Clinical, laboratory, and hemodynamic characteristics of the study cohort.

	PWH (*n* = 36)	People Without HIV (*n* = 36)	*p*-Value
Sociodemographic and Clinical Parameters			
Age (years)	53 (51–57.8)	54 (51–58.8)	0.852
BMI (kg/m^2^)	26.3 ± 2.1	26.7 ± 2.1	0.399
Waist circumference (cm)	99.5 ± 8.3	98 ± 7.7	0.438
Smoking, n (%)	27 (75)	24 (66.7)	0.437
Pack years, (n)	24.5 (0.5–35.8)	17 (0–34.5)	0.545
Physical activity, n (%)	18 (50)	13 (36.1)	0.234
Alcohol abuse, n (%)	0 (0)	1 (2.8)	1.000
Dyslipidemia, n (%)	21 (58.3)	23 (63.9)	0.629
Lipid-lowering agents, n (%)	8 (22.2)	7 (19.4)	0.772
FRS, (n)	15.6 (11.2–23.4)	13.2 (8.5–17.6)	0.107
HellenicSCORE2, (n)	3 (2–4)	3 (2–3.9)	0.416
Laboratory parameters			
Fasting glucose (mg/dl)	92 (87–98)	95 (84–104)	0.782
Total cholesterol (mg/dl)	194 ± 32	200 ± 36	0.477
Triglycerides (mg/dl)	134 ± 54	124 ± 55	0.439
HDL cholesterol (mg/dl)	45 ± 10	53 ± 12	0.002
LDL cholesterol (mg/dl)	122 ± 28	122 ± 32	0.960
eGFR (mL/min/1.73 m^2^)	90.4 ± 17.2	88.6 ± 16	0.638
Hemodynamic parameters			
bSBP (mmHg)	127.2 ±11.6	126.4 ± 14.1	0.799
bDBP (mmHg)	80.4 ± 6.7	78.7 ± 10.1	0.396
bPP (mmHg)	46.8 ± 8.4	47.7 ± 8.4	0.636
MBP (mmHg)	96 ± 7.7	94.6 ± 10.9	0.531
aSBP (mmHg)	116.8 ± 11.6	116.11 ± 13.3	0.836
aDBP (mmHg)	81.6 ± 6.8	79.8 ± 10.1	0.371
aPP (mmHg)	35.1 ± 7.7	36.4 ± 7.3	0.493
Heart rate (bpm)	74 ± 10	71 ± 10	0.222
AIx@75 (%)	21.6 ± 7.4	18.5 ± 8.2	0.092
cfPWV (m/s)	9.3 ± 1.4	8.6 ± 1.2	0.019

Quantitative data are presented as mean ± SD or median (25th–75th percentile), and categorical variables as number and percentages (n, %). Student’s *t*-test, Mann–Whitney U-test and chi-square test were used to compare means, medians, and differences in categorical data, respectively. BMI, body mass index; FRS, Framingham risk score; HDL, high density lipoprotein; LDL, low density lipoprotein; eGFR, estimated glomerular filtration rate; bSBP, brachial systolic blood pressure; bDBP, brachial diastolic blood pressure; bPP, brachial pulse pressure; MBP, mean blood pressure; aSBP, aortic systolic blood pressure; aDBP, aortic diastolic blood pressure; aPP, aortic pulse pressure; AIx@75, augmentation index adjusted to 75 beats per minute; cfPWV, carotid–femoral pulse wave velocity.

**Table 3:** HIV-related characteristics of PWH.

Variable	Value
Duration of HIV infection (years)	13.5 (8–19.8)
Duration of ART (years)	12 (7–18)
VL < 50 c/mL, n (%)	35 (97.2)
Current CD4+T-cell count (cells/mm^3^)	831 ± 365
Current CD4+T-cell < 200 cells/mm^3^ (%)	0 (0)
Nadir CD4+T-cell count (cells/mm^3^)	269 (111–368)
Nadir CD4+T-cell < 200 cells/mm^3^ (%)	14 (38.9)
D:A:D 5-year risk score	5.8 ± 2.9
Current NNRTI-based ART	
n (%)	13 (36.1)
Years	9.4 ± 3.1
Current PI-based ART	
n (%)	15 (41.7)
Years	11.9 ± 5.6
Current INSTI-based ART	
n (%)	15 (41.7)
Years	2 (1–4)
Current TDF-based ART	
n (%)	25 (69.4)
Years	6 ± 2.7
Current ABC-based ART	
n (%)	2 (5.6)
Years	6.5 ± 0.7
Cumulative NNRTI-based ART	
n (%)	19 (52.8)
Years	9.8 ± 4.2
Cumulative PI-based ART	
n (%)	26 (72.2)
Years	7 (6–14)
Cumulative INSTI-based ART	
n (%)	16 (44.4)
Years	2 (1–5)
Cumulative TDF-based ART	
n (%)	32 (88.9)
Years	6.5 ± 3
Cumulative ABC-based ART	
n (%)	7 (19.4)
Years	5.3 ± 3.1

Quantitative data are presented as mean ± SD or median (25th–75th percentile), and categorical variables as number and percentages (n, %). HIV, human immunodeficiency virus; ART, antiretroviral therapy; VL, viral load; CD4, cluster of differentiation 4; D:A:D, data collection on adverse events of anti-HIV drugs; NNRTI, nonnucleoside reverse transcriptase inhibitor; PI, protease inhibitor; INSTI, integrase inhibitor; TDF, tenofovir disoproxil; ABC, abacavir.

**Table 4:** Differences in relative serum expression (2^−ΔCt^) and fold changes (2^−ΔΔ^Ct) of circulating miRNAs in PWH compared to people without HIV.

miRNA	2^−ΔCt^		*p*-Value	Fold Change Comparing to People Without HIV	95% CI
	PWH	People Without HIV			
let-7b-5p	0.127860	0.024419	0.027	5.24	1.86~8.61
miR-19b-3p	0.060110	0.099174	0.049	0.61	0.20~1.01
miR-21-5p	0.360600	0.105762	<0.001	3.41	1.75~5.07
miR-29a-3p	0.009141	0.008749	0.074	1.04	0.42~1.67
miR-126-3p	0.037678	0.030660	0.019	1.23	0.64~1.82
miR-130a-3p	0.003910	0.005483	0.686	0.71	0.32~1.10
miR-145-5p	0.008211	0.005691	0.190	1.44	0.45~2.44
miR-181b-5p	0.006856	0.002813	0.191	2.44	0.64~4.24
miR-221-3p	0.013447	0.017244	0.846	0.78	0.35~1.21
miR-222-3p	0.009027	0.002730	0.002	3.31	1.10~5.51
miR-223-3p	0.131354	0.045253	0.115	2.90	0.81~4.99

Student’s *t*-test was used to compare means. miR, microRNA; PWH, people with HIV, CI: confidence interval.

**Table 5:** Bivariable and multivariable linear regression analysis of relative serum expression of let-7b-5p in PWH.

Characteristics	Bivariable			Multivariable		
	β	95%CI	*p*-Value	β	95%CI	*p*-Value
Sociodemographic and Clinical Parameters						
Age (years)	0.027	−0.013~0.067	0.181	-	-	-
BMI (kg/m^2^)	−0.050	−0.150~0.049	0.314	-	-	-
Lipid-lowering agents, n (%)	0.021	−0.482~0.525	0.932	-	-	-
Smoking, n (%)	−0.056	−0.539~0.427	0.815	-	-	-
Pack years, (n)	0.002	−0.006~0.010	0.606	-	-	-
FRS, (n)	0.024	−0.003~0.050	0.077	-	-	-
HellenicSCORE2, (n)	0.076	−0.043~0.196	0.204	-	-	-
Laboratory Parameters						
Fasting glucose (mg/dl)	−0.004	−0.022~0.014	0.616	-	-	-
Total cholesterol (mg/dl)	0.003	−0.003~0.010	0.302	-	-	-
LDL cholesterol (mg/dl)	0.001	−0.006~0.008	0.760	-	-	-
eGFR (mL/min/1.73 m^2^)	0.002	−0.011~0.014	0.769	-	-	-
Hemodynamic Parameters						
bSBP (mmHg)	0.027	0.012~0.043	0.001	-	-	-
bDBP (mmHg)	0.024	−0.007~0.054	0.121	-	-	-
bPP (mmHg)	0.037	0.015~0.059	0.002	0.030	0.008~0.051	0.009
MBP (mmHg)	0.033	0.008~0.058	0.012	-	-	-
aSBP (mmHg)	0.025	0.009~0.041	0.003	-	-	-
aDBP (mmHg)	0.023	−0.007~0.053	0.132	-	-	-
aPP (mmHg)	0.040	0.016~0.063	0.002	-	-	-
AIx@75 (%)	0.012	−0.017~0.040	0.401	-	-	-
cfPWV (m/s)	0.120	−0.027~0.268	0.107	-	-	-
HIV-Related Parameters						
Duration of HIV infection (years)	0.006	−0.027~0.039	0.714	-	-	-
Duration of ART (years)	0.014	−0.019~0.046	0.398	-	-	-
Current CD4+T-cell count (cells/mm^3^)	0.001	−0.001~0.001	0.364	-	-	-
Nadir CD4+T-cell count (cells/mm^3^)	−0.001	−0.002~0.001	0.183	-	-	-
Nadir CD4+T-cell <200 cells/mm^3^ (%)	0.412	0.008~0.817	0.046	0.344	0.001~0.691	0.049
D:A:D 5-year score	0.033	−0.039~0.106	0.359	-	-	-
Current NNRTI-based ART	−0.184	−0.615~0.247	0.391	-	-	-
Current PI-based ART	0.253	−0.162~0.669	0.223	-	-	-
Current INSTI-based ART	0.280	−0.133~0.694	0.177	-	-	-
Current TDF-based ART	−0.519	−0.936~−0.102	0.016	−0.315	−0.701~0.072	0.107

PWH, people with HIV; CI, confidence interval; BMI, body mass index; FRS, Framingham risk score; LDL, low-density lipoprotein; eGFR, estimated glomerular filtration rate; bSBP, brachial systolic blood pressure; bDBP, brachial diastolic blood pressure; bPP, brachial pulse pressure; MBP, mean blood pressure; aSBP, aortic systolic blood pressure; aDBP, aortic diastolic blood pressure; aPP, aortic pulse pressure; AIx@75, augmentation index adjusted to 75 beats per minute; cfPWV, carotid-femoral pulse wave velocity; ART, antiretroviral therapy; CD4, cluster of differentiation 4; D:A:D, data collection on adverse events of anti-HIV drugs; NNRTI, nonnucleoside reverse transcriptase inhibitor; PI, protease inhibitor; INSTI, integrase inhibitor; TDF, tenofovir disoproxil.

**Table 6:** Bivariable and multivariable linear regression analysis of relative serum expression of miR-126-3p in PWH.

Characteristics	Bivariable			Multivariable		
	β	95%CI	*p*-Value	β	95%CI	*p*-Value
Sociodemographic and Clinical Parameters						
Age (years)	0.037	0.004~0.070	0.029	0.032	0.004~0.060	0.027
BMI (kg/m^2^)	0.020	−0.067~0.107	0.644	-	-	-
Lipid-lowering agents, n (%)	−0.016	−0.441~0.430	0.979	-	-	-
Smoking, n (%)	−0.056	−0.539~0.427	0.815	-	-	-
Pack years, (n)	−0.104	−0.520~0.313	0.616	-	-	-
FRS, (n)	−0.001	−0.008~0.005	0.662	-	-	-
HellenicSCORE2, (n)	0.075	−0.028~0.178	0.148	-	-	-
Laboratory Parameters						
Fasting glucose (mg/dl)	0.009	−0.006~0.024	0.237	-	-	-
Total cholesterol (mg/dl)	0.001	−0.006~0.006	0.945	-	-	-
LDL cholesterol (mg/dl)	0.001	−0.007~0.006	0.883	-	-	-
eGFR (mL/min/1.73 m^2^)	−0.014	−0.023~−0.004	0.007	−0.012	−0.020~−0.004	0.004
Hemodynamic Parameters						
bSBP (mmHg)	0.019	0.005~0.034	0.009	0.012	−0.001~0.024	0.066
bDBP (mmHg)	0.027	0.001~0.052	0.042	-	-	-
bPP (mmHg)	0.028	0.007~0.050	0.012	-	-	-
MBP (mmHg)	0.020	−0.001~0.041	0.060	-	-	-
aSBP (mmHg)	0.020	0.006~0.035	0.006	-	-	-
aDBP (mmHg)	0.027	0.001~0.052	0.040	-	-	-
aPP (mmHg)	0.026	0.003~0.048	0.025	-	-	-
AIx@75 (%)	0.010	−0.015~0.035	0.417	-	-	-
cfPWV (m/s)	0.060	−0.071~0.191	0.361	-	-	-
HIV-Related Parameters						
Duration of HIV infection (years)	−0.005	−0.034~0.023	0.696	-	-	-
Duration of ART (years)	−0.001	−0.029~0.028	0.955	-	-	-
Current CD4+T-cell count (cells/mm^3^)	0.001	−0.001~0.001	0.420	-	-	-
Nadir CD4+T-cell count (cells/mm^3^)	0.001	−0.001~0.002	0.158	-	-	-
Nadir CD4+T-cell <200 cells/mm^3^ (%)	−0.209	−0.573~0.155	0.251	-	-	-
D:A:D 5-year score	0.052	−0.009~0.112	0.093	-	-	-
Current NNRTI-based ART	0.402	0.052~0.752	0.025	0.389	0.100~0.678	0.010
Current PI-based ART	−0.104	−0.470~0.261	0.565	-	-	-
Current INSTI-based ART	−0.184	−0.545~0.178	0.308	-	-	-
Current TDF-based ART	−0.037	−0.430~0.356	0.849	-	-	-

PWH, people with HIV; CI, confidence interval; BMI, body mass index; FRS, Framingham risk score; LDL, low-density lipoprotein; eGFR, estimated glomerular filtration rate; bSBP, brachial systolic blood pressure; bDBP, brachial diastolic blood pressure; bPP, brachial pulse pressure; MBP, mean blood pressure; aSBP, aortic systolic blood pressure; aDBP, aortic diastolic blood pressure; aPP, aortic pulse pressure; AIx@75, augmentation index adjusted to 75 beats per minute; cfPWV, carotid-femoral pulse wave velocity; ART, antiretroviral therapy; CD4, cluster of differentiation 4; D:A:D, data collection on adverse events of anti-HIV drugs; NNRTI, nonnucleoside reverse transcriptase inhibitor; PI, protease inhibitor; INSTI, integrase inhibitor; TDF, tenofovir disoproxil.

## Data Availability

All data generated in this study are included in this published article. Further inquiries can be directed to the corresponding author.
